# Differential Expression of Lipid Metabolism-Related Proteins in Different Breast Cancer Subtypes

**DOI:** 10.1371/journal.pone.0119473

**Published:** 2015-03-09

**Authors:** Sewha Kim, YuKyung Lee, Ja Seung Koo

**Affiliations:** Department of Pathology, Yonsei University College of Medicine, 50 Yonsei-ro, Seodaemun-gu, Seoul 120-752, Republic of Korea; University Medical Center Hamburg-Eppendorf, GERMANY

## Abstract

**Purpose:**

This study aimed to determine the expression and clinical significance of proteins that are involved in lipid metabolism in human breast tumors.

**Methods:**

Tumors from 476 breast cancer patients were used to construct tissue microarrays. Then, immunohistochemistry (IHC) for hormone-sensitive lipase (HSL), Perilipin 1 (PLIN1), fatty acid binding protein 4 (FABP4), carnitine palmitoyltransferase IA (CPT-1A), acyl-CoA oxidase 1 (ACOX-1), and fatty acid synthase (FASN) was performed on these microarrays.

**Results:**

Breast tumors were classified into 4 subtypes: luminal A (*n* = 242; 50.8%), luminal B (*n* = 134; 28.2%), human epidermal growth factor receptor 2 (HER2) (*n* = 50; 10.5%), and triple negative breast cancer (TNBC) (*n* = 50; 10.5%). The expression of PLIN1 (*p* < 0.001), FABP4 (*p* = 0.029), CPT-1A (*p* = 0.001), ACOX-1 (*p* < 0.001), and FASN (*p* < 0.001) differed significantly among these tumor subtypes. Notably, PLIN1, CPT-1A, and FASN expression was highest in HER2 tumors and lowest in TNBC tumors. Similarly, the expression of FABP4 and ACOX-1 was highest in HER2 tumors and lowest in luminal A tumors. In addition, ACOX-1 positivity was associated with significantly shorter overall survival (*p* = 0.018). When tumor subtype was considered, FABP4 positivity was associated with significantly shorter disease-free survival (*p* = 0.005) and overall survival (*p* = 0.041) in TNBC.

**Conclusion:**

Lipid metabolism-related proteins are differentially expressed in different IHC subtypes of breast cancer and some are associated with decreased survival rates.

## Introduction

In oncology, the Warburg effect describes a significant metabolic change in energy production from oxidative phosphorylation in normal cells to aerobic glycolysis in cancer cells [1]. However, this definition is somewhat simplistic as tumors can use several different metabolic mechanisms to produce energy, depending on the type of tumor [2], which complicates targeted delivery of metabolic inhibitors to cancer cells. One such mechanism is lipid metabolism, which involves lipid synthesis, lipid degradation and catabolism, and fatty acid (FA) oxidation. Lipid synthesis includes FA and triacylglycerol (TAG) synthesis by fatty acid synthase (FASN) [3], and lipid degradation and catabolism include TAG, cholesterol ester, and phospholipid hydrolysis. A key enzyme in this process is hormone-sensitive lipase (HSL) [4–6]. FA oxidation involves catabolism of free fatty acids in the mitochondria to produce energy. Carnitine palmitoyltransferase IA (CPT-1A) and acyl-CoA oxidase 1 (ACOX-1) are two important enzymes in this process [5–7]. Aside from this process, lipid transport and uptake are indeed an important and under-appreciated aspect of lipid metabolism in cancer [8,9]. Two important proteins in this process are fatty acid binding protein 4 (FABP4), which transports free fatty acids, and Perilipin 1 (PLIN1), which helps to regulate triacylglycerol storage by suppressing its hydrolysis [10]. In addition, it is also becoming clear that lipid droplets are more than just passive storage components and are important in cancer as well, in particular for survival under stressful conditions [11,12], where lipid droplet proteins (HSL and PLIN1) play an important role.

Due to the clinical, histological, and molecular heterogeneity of breast tumors, many classification schemes have been proposed to group tumors with similar features. For example, gene profiling analyses of breast tumors have suggested 5 molecular subtypes, namely, luminal A, luminal B, HER2, normal breast-like, and basal-like) [13]. Since these subtypes differ in terms of their histology, clinical behavior, and therapeutic response, it is not surprising that they use metabolic pathways differentially. Indeed, previous studies have shown that proteins that are involved in glycolysis [14,15], glutaminolysis [16], and glycine or serine metabolism [17] are differentially expressed among different tumor subtypes. However, little is known about the differential expression of proteins that are involved in lipid metabolism in different breast cancer subtypes. As a result, this study aimed to determine the expression and clinical significance of proteins that are involved in lipolysis and mitochondrial β-oxidation in different breast cancer subtypes.

## Methods

### Cell culture and western blot

Five breast cancer cell lines, namely, MCF-7, MDA-MB-453, MDA-MB-435S, MDA-MB-231, and MDA-MB-468, were obtained from the American Type Culture Collection (ATCC). MDA-MB-435S, MDA-MB-231, and MDA-MB-468 cells were grown in Dulbecco’s modified Eagle’s medium (DMEM) supplemented with 10% fetal bovine serum (FBS) and 1% penicillin-streptomycin (Hyclone) in a humidified incubator with 5% CO_2_ at 37°C. MCF-7 cells were cultured in DMEM without phenol red (Gibco) supplemented with 10% FBS, 1% penicillin-streptomycin, and 10 mg/mL insulin. MDA-MB-453 cells were maintained in L-15 medium (ATCC) supplemented with 10% FBS.

Cells were harvested and lysed in RIPA buffer (50 mM Tris-HCl pH 7.4, 1% nonyl phenoxypolyethoxylethanol, 0.25% sodium deoxycholate, 150 mM NaCl, 1 mM ethylenediaminetetraacetic acid, and 0.1% sodium dodecyl sulfate [SDS]) containing protease inhibitors. Subsequently, lysates were centrifuged at 13,000 *g* for 15 min at 4°C. Protein concentrations were measured by using the bicinchoninic acid assay (Thermo-Scientific). An equal amount of protein from each sample was separated by SDS polyacrylamide gel electrophoresis and then blotted onto nitrocellulose membranes (Bio-Rad). The membranes were blocked with 7% nonfat dry milk in phosphate-buffered saline with Tween 20, and then incubated with primary antibodies against HSL, PLIN1, FABP4, CPT-1A, ACOX-1, and β-actin (Table 1) for 1 hour at room temperature. Then, the membranes were incubated with a horseradish peroxidase-conjugated secondary antibody for 1 hour at room temperature. Finally, the bound antibodies were visualized by using an enhanced chemiluminescent reagent (GE Healthcare Life Sciences). All antibodies were purchased from Abcam.

**Table 1:** Source, clone, and dilution of used antibodies.

Antibody	Clone	Catalogue number	Antigen retrieval	Dilution	Company
*Molecular subtype-related*					
ER	SP1	RM-9101-S	Citric acid / microwave	1:100	Thermo Scientific, CA,
					USA
PR	PgR 636	M3569	Citric acid / microwave	1:50	DAKO, Denmark
HER2	Polyclonal	A0485	Citric acid / microwave	1:1500	DAKO, Denmark
Ki-67	MIB-1	M7240	Citric acid / microwave	1:150	DAKO, Denmark
*Lipolysis-related*			Citric acid / microwave		
HSL	Polyclonal	ab45422	Citric acid / microwave	1:100	Abcam, Cambridge, UK
PLIN1	Polyclonal	ab61682	Citric acid / microwave	1:100	Abcam, Cambridge, UK
FABP4	Polyclonal	ab13979	Citric acid / microwave	1:100	Abcam, Cambridge, UK
CPT-1	8F6AE9	ab128568	Citric acid / microwave	1:200	Abcam, Cambridge, UK
Acyl-CoA oxidase 1	Polyclonal	ab128549	Citric acid / microwave	1:50	Abcam, Cambridge, UK
FASN	EPR7466	ab128870	Citric acid / microwave	1:200	Abcam, Cambridge, UK

### Patients selection

The study group consisted of 476 patients who were diagnosed with invasive breast cancer and underwent surgical excision at Yonsei University Severance Hospital (Seoul, Korea) between January 2002 and December 2006. Patients who received preoperative hormonal therapy or neoadjuvant chemotherapy were excluded from the study. This study was approved by the Institutional Review Board of Yonsei University Severance Hospital.

Hematoxylin and eosin (H&E)-stained tumor sections were retrospectively reviewed by a breast pathologist who graded the tumor using the Nottingham grading system [18]. For each patient, age at the time of initial diagnosis, lymph node metastasis, tumor recurrence, distant metastasis, and survival were recorded.

### Construction of tissue microarrays

A representative area was selected from a H&E section and the corresponding area was marked on the surface of the paraffin-embedded tissue block. Then, a paraffin tissue punch was used to extract a 3 mm core sample from the selected area, which was placed into a 6 × 5 recipient block. Two tissue cores were extracted to reduce sampling bias. Each core was assigned a unique tissue microarray location number, which was linked to a database that contained other clinicopathological data.

### Immunohistochemistry

All immunohistochemical staining was performed on formalin-fixed, paraffin-embedded tissue sections. Briefly, 5 μm-thick sections were cut with a microtome, transferred onto adhesive slides, and then dried at 62°C for 30 minutes. All slides were incubated with primary antibodies (Table 1). After applying primary antibodies, blocking time was 2 hours at 37°C. Subsequently, immunodetection was performed by using a commercial streptavidin-biotin kit according to the manufacturer’s instructions, which involved incubation with biotinylated anti-mouse or anti-rabbit immunoglobulin, followed by peroxidase-labeled streptavidin and 3,3′-diaminobenzidine chromogenic substrate. The primary antibody incubation step was omitted in the negative control. Finally, the slides were counterstained with Harris hematoxylin.

### Interpretation of immunohistochemical staining

The status of all immunohistochemical markers was determined by using light microscopy to assess the fraction of stained cells. HSL, PLIN1, FABP4, CPT-1A, ACOX-1, and fatty acid synthase (FASN) immunostaining were scored as the product of the proportion of stained cells (0 = no staining, 1 = less than 30%, or 2 = more than 30%) and staining intensity (0 = no staining, 1 = weak, 2 = moderate, or 3 = strong). The scores for the proportion of stained cells and the staining were multiplied to provide a total score. A total score of 2–6 was considered positive, while a score of 0 or 1 was considered negative [19]. Similarly, Ki-67 immunostains were scored as the percentage of stained tumor cells, which was defined as the Ki-67 labeling index (LI). In addition, estrogen receptor (ER) and progesterone receptor (PR) positivity were defined as one percent or more cells having positively stained nuclei [20].

HER2 immunohistochemistry (IHC) results were classified according to American Society of Clinical Oncology/College of American Pathologists (ASCO/CAP) guidelines, which includes the following categories: 0 = no immunostaining; 1+ = weak/incomplete membrane staining in less than 10% of tumor cells; 2+ = complete membrane staining that is either uniform or weak in at least 10% of all tumor cells; and 3+ = uniform, intense membrane staining in at least 30% of tumor cells. HER2 positivity was defined as IHC 3+, while IHC 0 or 1+ were considered to be HER2 negative [21]. However, IHC 2+ is an equivocal classification, so in these cases, HER2 expression was further evaluated with fluorescence *in situ* hybridization (FISH).

### Fluorescence *in situ* hybridization (FISH) analysis

FISH was performed by using a PathVysion HER2 DNA Probe Kit (Vysis, Downers Grove, IL, USA) according to the manufacturer’s instructions. Then, the *HER2* gene copy number was quantified by using an epifluorescence microscope (Olympus, Tokyo, Japan). At least 60 tumor cell nuclei from three separate regions were used to measure signals from DNA probes specific for *HER2* and the centromeric region of chromosome 17 (CEP17). *HER2* gene amplification was determined according to ASCO/CAP guidelines [21]. Specifically, HER2 negativity was defined as a dual-probe HER2/CEP17 signal ratio less than 1.8 with an absolute *HER2* gene copy number of less than 4 signals per cell. On the other hand, a HER2/CEP17 ratio greater than 2.2 with an absolute *HER2* gene copy number of greater than 6 signals per cell was considered to be HER2 positive. HER2/CEP17 ratios between 1.8 and 2.2 or absolute *HER2* copy numbers between 4 and 6 were considered to be HER2 equivocal.

### Classification of tumor phenotypes

Breast tumor phenotypes were classified into four subtypes according to their IHC results for ER, PR, and Ki-67 and IHC/FISH results for HER2 as follows: (1) luminal A = ER+ and/or PR+, HER2-, and Ki-67 LI <14%; (2) luminal B = ER+ and/or PR+, HER2-, and Ki-67 LI ≥14%; or ER+ and/or PR+ and HER2+; (3) HER2 = ER-, PR-, and HER2+; and (4) triple negative breast cancer (TNBC) = ER-, PR-, and HER2− [22].

### Statistical analyses

Student’s *t* test and Fisher’s exact test were used to detect statistically significant differences (*p* < 0.05) in continuous and categorical variables, respectively. Kaplan-Meier survival curves and log-rank statistics were also used to estimate disease-free survival (DFS) and overall survival (OS) rates. Multivariate regression analysis was performed with the Cox proportional hazards model. All statistical analyses were calculated with SPSS for Windows, version 12.0 (SPSS Inc., Chicago, IL, USA).

## Results

### Patient characteristics

The clinicopathological characteristics of the 476 female patients in this study are shown in Table 2. The distribution of tumor subtypes was as follows: 242 luminal A (50.8%), 134 luminal B (28.2%), 50 HER2 (10.5%), and 50 TNBC (10.5%). When comparing clinicopathologic factors among molecular subtypes, TNBC tumors tended to have a higher histologic grade (*p* < 0.001) and higher Ki-67 LI (*p* < 0.001) than other subtypes.

**Table 2:** Clinicopathologic characteristics of patients according to breast cancer phenotype.

Parameter	Total	Luminal A	Luminal B	HER2	TNBC	*P*-value*
	(n = 476)	(n = 242)	(n = 134)	(n = 50)	(n = 50)	
	(100%)	(50.8%)	(28.2%)	(10.5%)	(10.5%)	
Age (Years, mean ±SD)	50.5±10.3	51.0±10.3	47.9±9.7	53.3±9.6	51.9±11.8	**0.003**
Histologic grade						**<0.001**
I/II	352 (73.9)	220 (90.9)	87 (64.9)	29 (58.0)	16 (32.0)	
III	124 (26.1)	22 (9.1)	47 (35.1)	21 (42.0)	34 (68.0)	
Tumor stage						0.577
T1	274 (57.6)	145 (59.9)	77 (57.5)	27 (54.0)	25 (50.0)	
T2/T3	202 (42.4)	97 (40.1)	57 (42.5)	23 (46.0)	25 (50.0)	
Nodal metastasis						0.288
Absent	283 (59.5)	139 (57.4)	78 (58.2)	30 (60.0)	36 (72.0)	
Present	193 (40.5)	103 (42.6)	56 (41.8)	20 (40.0)	14 (28.0)	
Estrogen receptor status						**<0.001**
Negative	110 (23.1)	5 (2.1)	5 (3.7)	50 (100.0)	50 (100.0)	
Positive	366 (76.9)	237 (97.9)	129 (96.3)	0 (0.0)	0 (0.0)	
Progesterone receptor status						**<0.001**
Negative	162 (34.0)	34 (14.0)	28 (20.9)	50 (100.0)	50 (100.0)	
Positive	314 (66.0)	208 (86.0)	106 (79.1)	0 (0.0)	0 (0.0)	
HER2 status						**<0.001**
Negative	349 (73.3)	242 (100.0)	57 (42.5)	0 (0.0)	50 (100.0)	
Positive	127 (26.7)	0 (0.0)	77 (57.5)	50 (100.0)	0 (0.0)	
Ki-67 LI (%)						**<0.001**
≤14	305 (64.1)	242 (100.0)	41 (30.6)	16 (32.0)	6 (12.0)	
>14	171 (35.9)	0 (0.0)	93 (69.4)	34 (68.0)	44 (88.0)	
Tumor recurrence	22 (4.6)	8 (3.3)	5 (3.7)	5 (10.0)	4 (8.0)	0.124
No. of patient deaths	26 (5.5)	9 (3.7)	5 (3.7)	5 (10.0)	7 (14.0)	**0.010**
Duration of clinical follow-	58.5±15.3	60.1±14.1	58.8±15.7	52.9±20.0	55.5±13.1	0.074
up (months, mean ± SD)						
TNBC, triple negative breast cancer.						

* *P*-value was calculated by Fisher’s exact test.

### Differential expression of lipid metabolism-related proteins in *in vitro* cell lines

**Fig 1:**
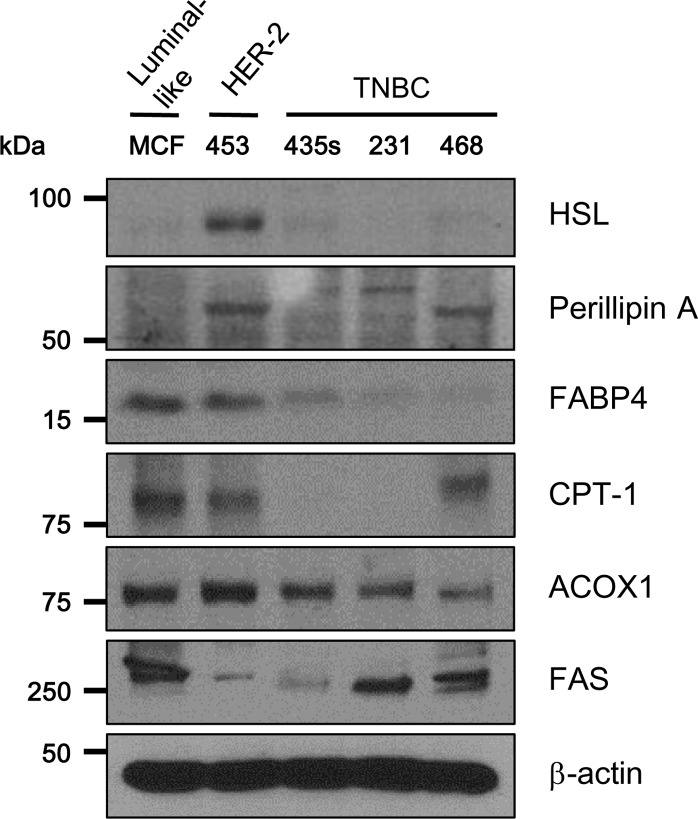
Expression of lipolysis-related proteins in five subtypes of breast cancer cells. MCF-7 (luminal-like), MDA-MB-453 (HER2), MDA-MB-435S (TNBC), MDA-MB-231 (TNBC), and MDA-MB-468 (TNBC) cells were lysed with RIPA buffer, and then cell lysates were subjected to SDS-PAGE and blotted with the indicated antibodies. Blots are representative of three independent experiments. TNBC, triple-negative breast cancer; HSL, hormone sensitive lipase; FABP4, fatty-acid binding protein 4; CPT-1A, carnitine palmitoyltransferase IA; ACOX1, Acyl-CoA oxidase 1; FASN, fatty acid synthase.

Western blotting revealed differential expression of lipid metabolism-related proteins among different breast cancer cell lines. Specifically, HSL and ACOX-1 were highly expressed in MDA-MB-453 cells (HER2 subtype). The expression of FABP4 and CPT-1A in MCF-7 cells (luminal subtype) and MDA-MB-453 cells was higher than in MDA-MB-435S, MDA-MB-231, and MDA-MB-468 cells (TNBC subtypes) (Fig. 1).

### Differential expression of lipid metabolism-related proteins in different tumor subtypes

**Fig 2:**
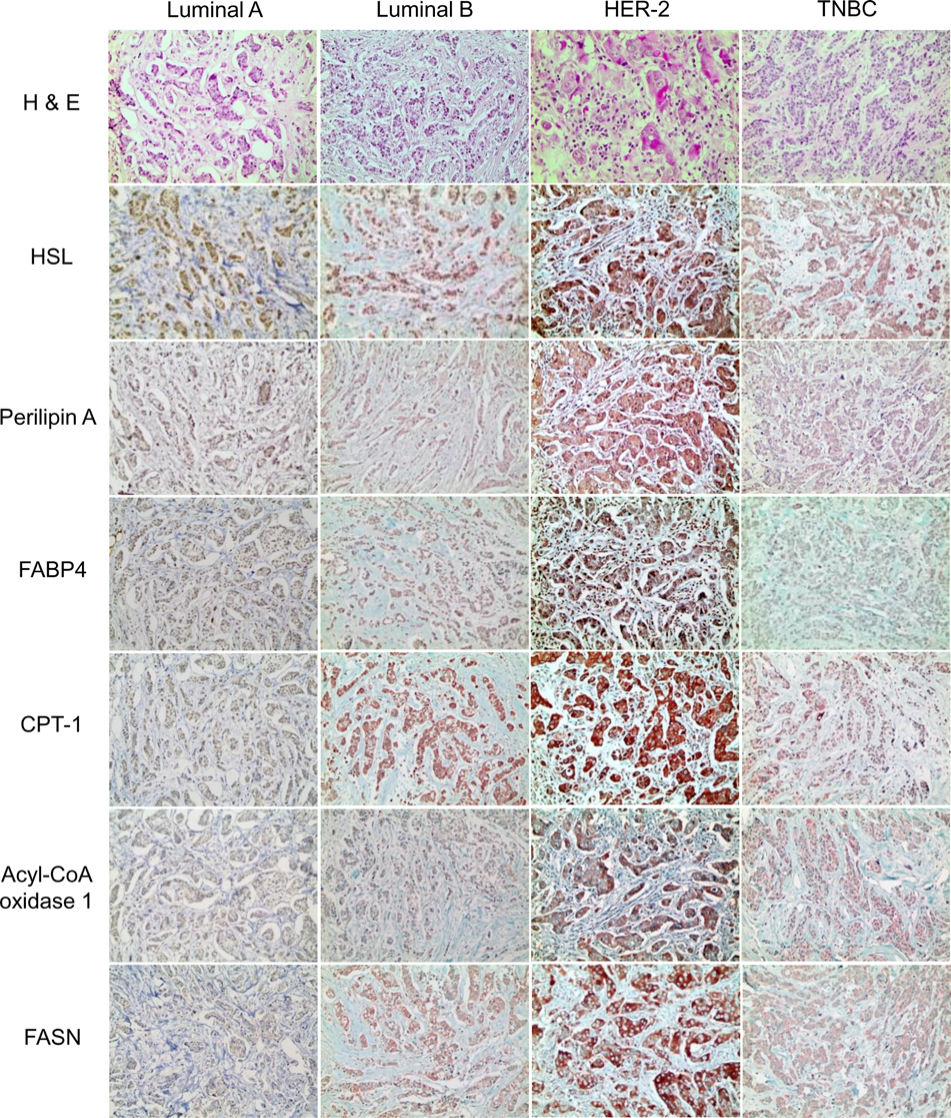
Expression of lipid metabolism related proteins according to the molecular subtypes of breast cancer. PLIN1, CPT-1A, and FASN expression are highest in HER2 tumors and lowest in TNBC tumors. Similarly, FABP4 and ACOX-1 expression are highest in HER2 tumors and lowest in luminal A tumors.

When comparing expressions of lipid metabolism—related proteins among molecular subtypes, the expression of PLIN1 (*p* < 0.001), FABP4 (*p* = 0.029), CPT-1A (*p* = 0.001), ACOX-1 (*p* < 0.001), and FASN (*p* < 0.001) differed significantly among the different tumor subtypes. Specifically, PLIN1, CPT-1A, and FASN expression were highest in HER2 tumors and lowest in TNBC tumors. Similarly, FABP4 and ACOX-1 expression were highest in HER2 tumors and lowest in luminal A tumors (Table 3 and Fig. 2).

**Table 3:** Expression of metabolism-related proteins according to breast cancer subtype.

Parameter	Total	Luminal A	Luminal B	HER2	TNBC	*P*-value*
	(n = 476) (%)	(n = 242) (%)	(n = 134) (%)	(n = 50) (%)	(n = 50) (%)	
HSL						0.082
Negative	406 (85.3)	199 (82.2)	115 (85.8)	44 (88.0)	48 (96.0)	
Positive	70 (14.7)	43 (17.8)	19 (14.2)	6 (12.0)	2 (4.0)	
PLIN1						**<0.001**
Negative	422 (88.7)	222 (91.7)	120 (89.6)	34 (68.0)	46 (92.0)	
Positive	54 (11.3)	20 (8.3)	14 (10.4)	16 (32.0)	4 (8.0)	
FABP4						**0.029**
Negative	468 (98.3)	240 (99.2)	133 (99.3)	47 (94.0)	48 (96.0)	
Positive	8 (1.7)	2 (0.8)	1 (0.7)	3 (6.0)	2 (4.0)	
CPT-1A						**0.001**
Negative	406 (85.3)	217 (89.7)	107 (79.9)	36 (72.0)	46 (92.0)	
Positive	70 (14.7)	25 (10.3)	27 (20.1)	14 (28.0)	4 (8.0)	
Acyl-CoA oxidase 1						**<0.001**
Negative	418 (87.8)	234 (96.7)	121 (90.3)	22 (44.0)	41 (82.0)	
Positive	58 (12.2)	8 (3.3)	13 (9.7)	28 (56.0)	9 (18.0)	
FASN						**<0.001**
Negative	312 (65.5)	160 (66.1)	88 (65.7)	22 (44.0)	42 (84.0)	
Positive	164 (34.5)	82 (33.9)	46 (34.3)	28 (56.0)	8 (16.0)	

TNBC, triple negative breast cancer.

* *P*-value was calculated by Fisher’s exact test.

### Correlation between expression of lipid metabolism-related proteins and clinicopathological characteristics

As shown in Table 4, HER2 positivity was significantly associated with CPT-1A positivity (*p* < 0.001) and ACOX-1 positivity (*p* < 0.001). ACOX-1 positivity also tended to be associated with a higher histologic grade (*p* = 0.006), ER negativity (*p* < 0.001), PR negativity (*p* < 0.001), and a higher Ki-67 LI (*p* < 0.001). FABP4 positivity was significantly correlated with ER negativity (*p* = 0.018).

**Table 4:** Correlations between the expression of lipid metabolism—related proteins and clinicopathologic parameters.

Parameters	HSL			PLIN1			FABP4		
	Negative	Positive	*P*-value*	Negative	Positive	*P*-value*	Negative	Positive	*P*-value*
	n = 406 (%)	n = 70 (%)		n = 422 (%)	n = 54 (%)		n = 468 (%)	n = 8 (%)	
Age (Years, mean ±SD)	50.6±10.3	49.4±10.5	2.232	50.3±10.2	52.1±11.1	1.380	50.5±10.3	46.2±11.7	1.458
Histologic grade			3.060			0.186			0.186
I/II	298 (73.4)	54 (77.1)		319 (75.6)	33 (61.1)		349 (74.6)	3 (37.5)	
III	108 (26.6)	16 (22.9)		103 (24.4)	21 (38.9)		119 (25.4)	5 (62.5)	
Tumor stage			2.874			0.906			1.482
T1	231 (56.9)	43 (61.4)		238 (56.4)	36 (66.7)		271 (57.9)	3 (37.5)	
T2/T3	175 (43.1)	27 (38.6)		184 (43.6)	18 (33.3)		197 (42.1)	5 (62.5)	
Nodal metastasis			4.296			3.462			5.160
Absent	240 (59.1)	43 (61.4)		249 (59.0)	34 (63.0)		278 (59.4)	5 (62.5)	
Present	166 (40.9)	27 (38.6)		173 (41.0)	20 (37.0)		190 (40.6)	3 (37.5)	
Estrogen receptor status			0.168			0.060			0.018
Negative	101 (24.9)	9 (12.9)		90 (21.3)	20 (37.0)		104 (22.2)	6 (75.0)	
Positive	305 (75.1)	61 (87.1)		332 (78.7)	34 (63.0)		364 (77.8)	2 (25.0)	
Progesterone receptor status			0.096			0.018			0.126
Negative	147 (36.2)	15 (21.4)		134 (31.8)	28 (51.9)		156 (33.3)	6 (75.0)	
Positive	259 (63.8)	55 (78.6)		288 (68.2)	26 (48.1)		312 (66.7)	2 (25.0)	
HER2 status			2.604			0.078			1.308
Negative	295 (72.7)	54 (77.1)		317 (75.1)	32 (59.3)		345 (73.7)	4 (50.0)	
Positive	111 (27.3)	16 (22.9)		105 (24.9)	22 (40.7)		123 (26.3)	4 (50.0)	
Ki-67 LI (%)			0.168			0.132			0.858
≤14	252 (62.1)	53 (75.7)		278 (65.9)	27 (50.0)		302 (64.5)	3 (37.5)	
>14	154 (37.9)	17 (24.3)		144 (34.1)	27 (50.0)		166 (35.5)	5 (62.5)	
Tumor recurrence	21 (5.2)	1 (1.4)	1.356	20 (4.7)	2 (3.7)	6.000	21 (4.5)	1 (12.5)	1.902
Patient death	24 (5.9)	2 (2.9)	2.412	24 (5.7)	2 (3.7)	4.530	25 (5.3)	1 (12.5)	2.184

Parameters	CPT-1			Acyl-CoA oxidase 1			FASN		
	Negative	Positive	*P*-value*	Negative	Positive	*P*-value*	Negative	Positive	*P*-value*
	n = 406 (%)	n = 70 (%)		n = 504 (%)	n = 236 (%)		n = 504 (%)	n = 236 (%)	
Age (Years, mean ±SD)	50.4±10.2	50.8±11.1	4.806	50.3±10.5	51.7±8.8	1.896	51.6±10.5	48.4±9.8	0.012
Histologic grade			3.618			<0.001			0.060
I/II	302 (74.4)	50 (71.4)		321 (76.8)	31 (53.4)		219 (70.2)	133 (81.1)	
III	104 (25.6)	20 (28.6)		97 (23.2)	27 (46.6)		93 (29.8)	31 (18.9)	
Tumor stage			4.410			2.994			4.704
T1	235 (57.9)	39 (55.7)		243 (58.1)	31 (53.4)		181 (58.0)	93 (56.7)	
T2/T3	171 (42.1)	31 (44.3)		175 (41.9)	27 (46.6)		131 (42.0)	71 (43.3)	
Nodal metastasis			0.486			3.990			4.614
Absent	248 (61.1)	35 (50.0)		247 (59.1)	36 (62.1)		184 (59.0)	99 (60.4)	
Present	158 (38.9)	35 (50.0)		171 (40.9)	22 (37.9)		128 (41.0)	65 (39.6)	
Estrogen receptor status			1.440			<0.001			5.892
Negative	90 (22.2)	20 (28.6)		72 (17.2)	38 (65.5)		72 (23.1)	38 (23.2)	
Positive	316 (77.8)	50 (71.4)		346 (82.8)	20 (34.5)		240 (76.9)	126 (76.8)	
Progesterone receptor status			0.942			<0.001			5.208
Negative	133 (32.8)	29 (41.4)		122 (29.2)	40 (69.0)		107 (34.3)	55 (33.5)	
Positive	273 (67.2)	41 (58.6)		296 (70.8)	18 (31.0)		205 (65.7)	109 (66.5)	
HER2 status			<0.001			<0.001			0.432
Negative	310 (76.4)	39 (55.7)		328 (78.5)	21 (36.2)		237 (76.0)	112 (68.3)	
Positive	96 (23.6)	31 (44.3)		90 (21.5)	37 (63.8)		75 (24.0)	52 (31.7)	
Ki-67 LI (%)			2.652			<0.001			4.962
≤14	263 (64.8)	42 (60.0)		282 (67.5)	23 (39.7)		201 (64.4)	104 (63.4)	
>14	143 (35.2)	28 (40.0)		136 (32.5)	35 (60.3)		111 (35.6)	60 (36.6)	
Tumor recurrence	18 (4.4)	4 (5.7)	3.288	18 (4.3)	4 (6.9)	1.968	18 (5.8)	4 (2.4)	0.678
Patient death	19 (4.7)	7 (10.0)	0.510	20 (4.8)	6 (10.3)	0.678	21 (6.7)	5 (3.0)	0.816

* *P*-values are corrected for multiple testing using the Bonferroni correction.

### Association of the expression of lipid metabolism—related proteins with prognosis

**Fig 3:**
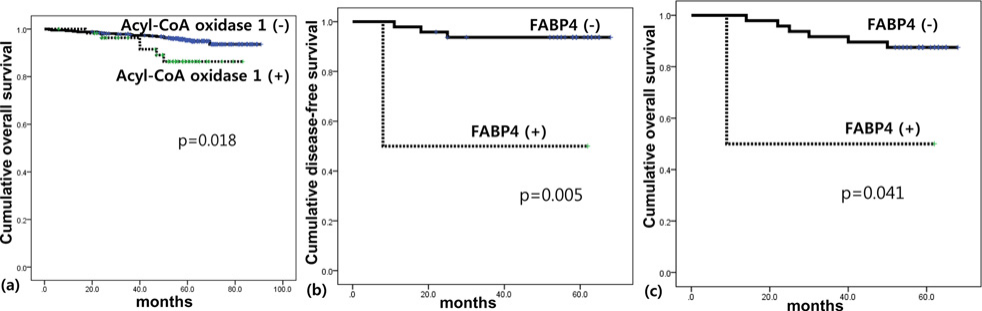
Impact of the expression of lipid metabolism related proteins on patient prognosis in breast cancer (a) and triple negative breast cancer (b,c).

The only statistically significant relationship revealed by univariate analysis was an association between ACOX-1 positivity and shorter OS (*p* = 0.018; Table 5). However, when tumor subtype was considered, there were also significant associations between FABP4 positivity and shorter DFS (*p* = 0.005) and shorter OS (*p* = 0.041) in TNBC (Fig. 3).

**Table 5:** The impact of expression of lipid metabolism-related proteins on prognosis.

Parameter	Number of patients/recurrence/death	Disease-free survival		Overall survival	
		Mean survival	*P*-value	Mean survival	*P*-value
		(95% CI) months		(95% CI) months	
HSL			0.176		0.315
Negative	406/21/24	87 (85–88)		87 (85–88)	
Positive	70/1/2	84 (83–85)		83 (81–85)	
PLIN1			0.795		0.632
Negative	422/20/24	87 (86–89)		87 (85–88)	
Positive	54/2/2	81 (78–84)		81 (78–84)	
FABP4			0.161		0.233
Negative	468/21/25	87 (86–89)		87 (86–88)	
Positive	8/1/1	59 (43–74)		59 (44–74)	
CPT-1A			0.608		0.069
Negative	406/18/19	87 (86–89)		87 (86–89)	
Positive	70/4/7	85 (81–89)		83 (79–87)	
Acyl-CoA oxidase 1			0.184		**0.018**
Negative	418/18/20	87 (86–89)		87 (86–89)	
Positive	58/4/6	78 (73–82)		77 (72–81)	
FASN			0.111		0.106
Negative	312/18/21	86 (84–88)		86 (84–88)	
Positive	164/4/5	87 (86–89)		87 (85–88)	

Multivariate Cox regression analysis revealed that higher T stage is an independent predictive factor for shorter DFS (hazard ratio: 3.801, 95% CI: 1.361–10.61, *p* = 0.011), but no other parameter had a statistically significant association with either DFS or OS (Table 6).

**Table 6:** Multivariate analysis of breast cancer survival.

Included parameters	Disease-free survival			Overall survival		
	Hazard ratio	95% CI	*P*-value	Hazard ratio	95% CI	*P*-value
T stage			**0.011**			0.107
T1 versus T2–3	3.801	1.361–10.61		2.021	0.859–4.752	
N stage			0.183			0.911
N0 versus N1–3	1.848	0.748–4.565		0.953	0.413–2.202	
Age			0.731			0.476
<50 versus ≥50	1.167	0.483–2.823		0.742	0.327–1.686	
Histologic grade			0.463			0.946
I/II versus III	1.467	0.527–4.083		1.035	0.383–2.797	
ER status			0.272			0.304
Negative versus Positive	1.974	0.587–6.641		1.857	0.571–6.043	
PR status			0.234			0.146
Negative versus Positive	1.989	0.641–6.170		2.240	0.756–6.641	
HER2 status			0.979			0.459
Negative versus Positive	1.014	0.360–2.858		0.688	0.256–1.849	
Ki-67 LI			0.242			0.595
≤14 versus >14	0.540	0.191–1.525		0.768	0.290–2.031	
HSL			0.399			0.595
Negative versus Positive	0.410	0.051–3.267		0.663	0.146–3.014	
PLIN1			0.930			0.672
Negative versus Positive	1.072	0.227–5.071		0.716	0.152–3.367	
FABP4			0.319			0.276
Negative versus Positive	2.970	0.349–25.29		3.270	0.387–27.61	
CPT1			0.846			0.072
Negative versus Positive	1.125	0.343–3.686		2.406	0.924–6.262	
Acyl-CoA oxidase 1			0.647			0.161
Negative versus Positive	1.342	0.381–4.730		2.180	0.733–6.481	
FASN			0.164			0.076
Negative versus Positive	0.438	0.137–1.402		0.371	0.124–1.109	

**Fig 4:**
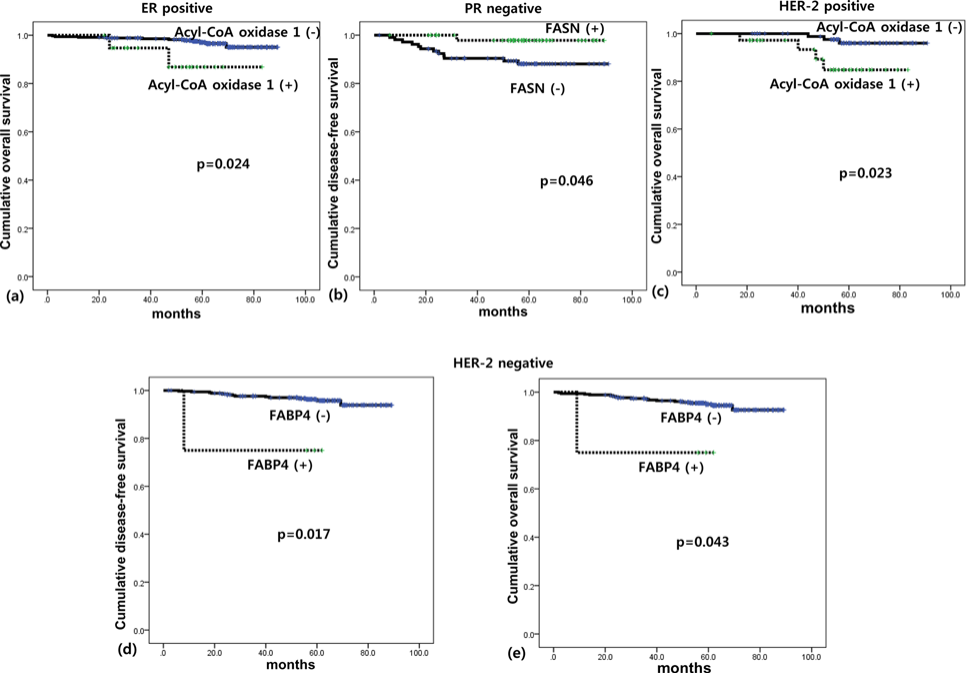
Impact of the expression of lipid metabolism related proteins on patient prognosis in ER positive group (a), PR negative group (b), HER2 positive group (c), and HER2 negative group (d, e).

The effect of the expression of lipid metabolism-related proteins on survival rates depending on ER, PR, and HER2 status of breast tumors is shown in Fig. 4. ACOX-1 positivity was associated with shorter OS in patients with ER positive tumors (*p* = 0.024) and HER2 positive tumors (*p* = 0.023). In addition, FASN negativity was associated with shorter DFS in patients with PR negative tumors (*p* = 0.046).

## Discussion

In this study, we observed differential expression of lipid metabolism-related proteins in 4 breast cancer subtypes. In particular, HER2 tumors showed the highest expression of PLIN1, CPT-1A, FASN, FABP4, and ACOX-1. This finding is consistent with two previous studies about HER2 and FASN. In one study, FASN expression was strongly correlated with HER2 status [23]. Another study showed that HER2 interacts with FASN and promotes FASN phosphorylation, which increases its activity and leads to cancer cell proliferation, and eventually, metastasis [24]. Our results suggest that lipid metabolism in breast tumors with the HER2 subtype is higher than that in other subtypes. Previous studies showed the difference of lipid metabolism genes according to breast cancer subtypes: products of de novo fatty acid synthesis, such as palmitate-containing phosphatidylcholine, were high in ER negative and grade 3 breast cancers [25], and acyl-CoA:cholesterol acyltransferase 1 (ACAT) activity was high in ER-negative basal-like breast cancer [26]. However, there was one report that the gene for secreted phospholipase A2 (sPLA2) is silenced in triple negative breast cancer cells [27]. Therefore, further study is required to evaluate the status of lipid metabolism genes according to breast cancer subtypes.

More generally, our results support the hypothesis that different breast cancer subtypes use different metabolic pathways, which has been previously suggested [2]. In this study, the expression of three lipid metabolism-related proteins (PLIN1, CPT-1A, and FASN) in TNBC cells was lower than that in other subtypes. Although a previous study demonstrated that the gene for secreted phospholipase A2 (sPLA2) is silenced in triple negative breast cancer cells [27], another report showed that sPLA2-induced lipid droplet formation plays an important role in TNBC cell proliferation and survival during starvation [12], indicating controversial results. Specifically, TNBC is often associated with a high histologic grade, accelerated mitosis, tumor necrosis, aggressive clinical behavior, and poor prognosis [28,29]. In addition, glycolysis-related proteins are highly expressed in TNBC and basal-like breast tumors, which imply high glycolytic activity [14,15], suggesting high metabolic activity in TNBC. Therefore, further study of lipid metabolism activity in TNBC is required.

The preference of different breast cancer subtypes for different metabolic pathways may have clinical implications. For example, ACOX-1 positivity was significantly correlated with shorter OS as well as clinicopathological characteristics, such as higher histologic grade (*p* = 0.006), ER negativity (*p* < 0.001), PR negativity (*p* < 0.001), and higher Ki-67 LI (*p* < 0.001), that are associated with poor prognosis in breast cancer patients. These results are in accordance with a previous study that showed that ACOX-1 expression increases with worsening histologic grade in brain glioma [30]. Similarly, FABP4 positivity was significantly associated with shorter DFS and OS in TNBC patients. These findings are supported by the observations that serum FABP levels are significantly higher in breast cancer patients than in healthy patients, and high serum levels of FABP are associated with adverse tumor characteristics, such as large tumor size and lymph node metastasis [31]. Similar correlations have also been found in bladder cancer [32] and prostate cancer [33].

These differences suggest that selective inhibition of lipid metabolism-related proteins may be a potential chemotherapeutic strategy for some breast cancer subtypes. This hypothesis is supported by the observation that inhibitors of glycolysis-related molecules, such as hypoxia-inducible factor 1α [34,35], glucose transporter 1 [36,37], carbonic anhydrase IX [38], and monocarboxylate transporter 4 [39], suppress tumor progression in several types of cancer, including breast cancer. In addition, several FASN inhibitors have been shown to decrease tumor cell growth or increase tumor cell death [24,40,41]. However, further studies are needed to determine the value of lipid metabolism-related proteins as therapeutic targets.

In conclusion, our results showed that lipid metabolism-related proteins are differentially expressed in different breast cancer subtypes, which may aid the development of novel chemotherapeutic agents.
